# Association of Chronic Opioid Use With Presidential Voting Patterns in US Counties in 2016

**DOI:** 10.1001/jamanetworkopen.2018.0450

**Published:** 2018-06-22

**Authors:** James S. Goodwin, Yong-Fang Kuo, David Brown, David Juurlink, Mukaila Raji

**Affiliations:** 1 Department of Preventive Medicine and Community Health, University of Texas Medical Branch, Galveston; 2 Departments of Medicine, University of Texas Medical Branch, Galveston; 3 Sealy Center on Aging, University of Texas Medical Branch, Galveston; 4 University of Texas Medical Branch, Galveston; 5 Department of Medicine, University of Toronto, Toronto, Ontario, Canada; 6 Department of Pediatrics, University of Toronto, Toronto, Ontario, Canada; 7 Institute of Health Policy, Management and Evaluation, University of Toronto, Toronto, Ontario, Canada

## Abstract

**Importance:**

The causes of the opioid epidemic are incompletely understood.

**Objective:**

To explore the overlap between the geographic distribution of US counties with high opioid use and the vote for the Republican candidate in the 2016 presidential election.

**Design, Setting, and Participants:**

A cross-sectional analysis to explore the extent to which individual- and county-level demographic and economic measures explain the association of opioid use with the 2016 presidential vote at the county level, using rate of prescriptions for at least a 90-day supply of opioids in 2015. Medicare Part D enrollees (N = 3 764 361) constituting a 20% national sample were included.

**Main Outcomes and Measures:**

Chronic opioid use was measured by county rate of receiving a 90-day or greater supply of opioids prescribed in 2015.

**Results:**

Of the 3 764 361 Medicare Part D enrollees in the 20% sample, 679 314 (18.0%) were younger than 65 years, 2 283 007 (60.6%) were female, 3 053 688 (81.1%) were non-Hispanic white, 351 985 (9.3%) were non-Hispanic black, and 198 778 (5.3%) were Hispanic. In a multilevel analysis including county and enrollee, the county of residence explained 9.2% of an enrollee’s odds of receiving prolonged opioids after adjusting for individual enrollee characteristics. The correlation between a county’s Republican presidential vote and the adjusted rate of Medicare Part D recipients receiving prescriptions for prolonged opioid use was 0.42 (*P* < .001). In the 693 counties with adjusted rates of opioid prescription significantly higher than the mean county rate, the mean (SE) Republican presidential vote was 59.96% (1.73%), vs 38.67% (1.15%) in the 638 counties with significantly lower rates. Adjusting for county-level socioeconomic measures in linear regression models explained approximately two-thirds of the association of opioid rates and presidential voting rates.

**Conclusions and Relevance:**

Support for the Republican candidate in the 2016 election is a marker for physical conditions, economic circumstances, and cultural forces associated with opioid use. The commonly used socioeconomic indicators do not totally capture all of those forces.

## Key Points

**Question:**

To what extent do socioeconomic measurements explain the county-level association of the 2016 US Republican presidential vote with opioid use?

**Findings:**

This cross-sectional analysis of a national sample of Medicare claims data found that chronic use of prescription opioid drugs was correlated with support for the Republican candidate in the 2016 US presidential election. Individual and county-level socioeconomic measures explained much of the association between the presidential vote and opioid use.

**Meaning:**

The association of the presidential vote with chronic opioid use underscores the importance of cultural, economic, and environmental factors associated with the opioid epidemic.

## Teaser

This study examines US county-level correlations between the proportion of Medicare Part D enrollees receiving a ≥90-day supply of opioids and the proportion of the county voting for Donald Trump for US president.

## Introduction

The epidemic of opioid use is a public health crisis in the United States and other countries.^1,2^ Much of the use is fueled by physician prescribing habits, with about half of opioid-related deaths caused by prescription opioids.^2^

One notable finding is the marked geographic variation in opioid prescribing,^3,4,5,6,7,8^ which is closely mirrored by similar variations in deaths from opioids.^1^ Geographic variation persists after controlling for individual-level risk factors.^5^ A number of studies have described state and county characteristics associated with high opioid use. These include education, racial/ethnic composition, health care utilization, physician supply, percentage uninsured, percentage on Medicaid, poverty, income inequality, and rural vs urban setting.^6,9,10,11,12^ In general, these characteristics explain about one-third of the geographic variation.

In examining the maps showing the geographic distribution of the opioid epidemic, several observers have noted the similarity to the results of the 2016 presidential election.^13,14,15,16^ Counties and states with the highest opioid use were often areas carried by the Republican candidate in the election. This is not surprising, because aspects of the narrative analyzing the presidential vote echoed themes that occur in explanations for high opioid use.^17,18^ In particular, both sets of explanations emphasized economic stressors and the sense of being left out. This study examines the association at the county level between the rate of Medicare Part D enrollees receiving prescriptions for prolonged opioid use and the percentage of votes for the Republican candidate in the 2016 election. Of particular interest was the extent to which county-level indicators of socioeconomic status explained this association. Economic stressors are only partially captured by standard measures such as poverty rate or income inequality.^9^ Thus, we hypothesized that controlling for available demographic and economic indicators at the individual and county level would only partly explain the associations of chronic opioid use and the presidential vote.

## Methods

This study followed the Strengthening the Reporting of Observational Studies in Epidemiology (STROBE) reporting guideline for reporting cross-sectional studies.^19^ The sources of data included Medicare files, the American Community Survey (ACS) from the US Census Bureau, and presidential voting data from uselectionatlas.org. Medicare claims included enrollment and claims data for a 20% national sample of Medicare beneficiaries enrolled in 2015. Medicare beneficiaries who had complete Parts A, B, and D coverage and who were not enrolled in a health maintenance organization in 2014 were selected; beneficiaries who survived through April 1, 2015, were included in the analyses. Part D provides prescription drug coverage, and in 2015 approximately 72% of Medicare beneficiaries were enrolled.^20^ The National Drug Code, product name, therapeutic class description, and US Drug Enforcement Administration class code from the 2015 Red Book Select database were used to identify prescriptions for any opioid and for any insulin (used as a control). Chronic opioid use was defined as receiving a prescription for a 90-day or greater supply in 1 year.^8^ The University of Texas Medical Branch Institutional Review Board approved the study and waived any informed consent requirement because the research used deidentified data.

Medicare enrollment files provided information on patient age, sex, race/ethnicity, and original entitlement. A Medicaid indicator in the enrollment file was a proxy for low income. The Elixhauser Comorbidity Index was used to generate comorbidity measures for each enrollee from all claims in 2014 and categorized enrollees by number of comorbidities: 0, 1, 2, and 3 or more.

County levels of education, household income, unemployment, single household, and marital status were from the 2011 to 2015 ACS 5-year estimates. Rurality was measured by Rural-Urban Continuum Codes.^21^ Religious attendance information came from the 2010 US Religion Census. The 2016 presidential and congressional voting data came from uselectionatlas.org. The 2015 20% sample of Medicare member data was used to estimate the percentage of individuals in each county who were white, male, eligible for Medicaid, covered by a health maintenance organization, covered by Medicare Part D, and whose original entitlement resulted from a disability. There are 3142 counties or county equivalents according to the 2011 to 2015 ACS 5-year estimates. In Medicare sample data, there were 3128 counties or county equivalents with any Medicare enrollees. After excluding counties with fewer than 12 enrollees, there were 3118 counties. The 2016 voting data from uselectionatalas.org included data from 3101 counties. It did not include data from Alaska. The analyses include between 3100 and 3118 counties, depending on the analysis. State laws regulating opioid prescribing have been categorized into 7 groups by the Centers for Disease Control and Prevention^22^: (1) requires evaluation for substance use disorder and physician examination before prescribing; (2) requires continuing education in prescribing controlled substances, pain management, and identifying substance use disorders for all practitioners; (3) requires the use of a prescription drug monitoring program when prescribing opioids; (4) sets certain prescription drug limits for schedule III opioids; (5) regulates and imposes strict oversight of pain management clinics and pain treatment facilities; (6) requires written consent or treatment plan for treatment of chronic pain; and (7) requires or recommends consultation with a specialist (pain, psychiatry, addiction, or mental health) in certain circumstances.^22^

Maps were first created showing unadjusted county rates of receiving prescriptions for a 90-day or greater supply of opioids in 2015 among Medicare Part D enrollees and also the 2016 county presidential vote using ArcGIS geographic information system version 9.3 (Esri). Pearson correlations were generated between county rates of opioid use and county presidential vote. Then county rates of Medicare Part D enrollees who received prescriptions for a 90-day or greater supply of opioids in 2015 were generated, adjusted for age, original reason for Medicare enrollment (age 65 years, disability, or end-stage renal disease), sex, race/ethnicity, Medicaid eligibility, and number of comorbidities in a hierarchical generalized linear mixed model with county as a random effect. The intraclass correlation coefficient was estimated from a null model with no enrollee characteristics and also from the full model. The adjusted county rates and corresponding 95% conﬁdence intervals were calculated, ranked, and plotted. To assess how much the association of presidential voting with opioid use was explained by other county characteristics, 3 regression models were built with the adjusted county opioid rate as the dependent variable. The first model included only the percentage of the county vote for the Republican presidential candidate. The second model adjusted for county demographic characteristics and the third model added whether the state had any of 7 categories of laws regulating opioid prescribing.^22,23,24^ The parameter estimate and partial *R*^2^ for each characteristic were reported. All tests of statistical significance were 2-sided with significance set at *P* < .05, and analyses were performed with SAS Enterprise statistical software version 7.12 at the CMS Virtual Research Data Center (SAS Institute Inc).

## Results

The characteristics of the 20% sample of Medicare Part D enrollees are presented in Table 1. Of the 3 764 361 enrollees, 679 314 (18.0%) were younger than 65 years, 2 283 007 (60.6%) were female, 3 053 688 (81.1%) were non-Hispanic white, 351 985 (9.3%) were non-Hispanic black, and 198 778 (5.3%) were Hispanic; 999 912 enrollees (26.5%) received their original Medicare entitlement because of disability and 2 735 152 (72.7%) because they had reached age 65 years.

**Table 1.** Characteristics Associated With Chronic Opioid Use Among Medicare Part D Enrollees in 2015^a^

Enrollee Characteristics	Enrollees, No. (%) (N = 3 764 361)	Enrollees With ≥90-d Prescription for Opioids, No. (%) (n = 522 180)^a^		OR (95% CI)
Original reason for entitlement (age <65 y)				
Disabled	657 010 (17.4)	175 897 (26.8)		3.10 (3.08-3.13)
ESRD	22 304 (0.6)	5266 (23.6)		1.84 (1.78-1.90)
Original reason for entitlement (age ≥65 y)				
Reached age 65 y	2 735 152 (72.7)	249 873 (9.1)		1 [Reference]
Disabled	342 902 (9.1)	89 911 (26.2)		2.72 (2.70-2.75)
ESRD	6994 (0.2)	1233 (17.6)		1.37 (1.29-1.46)
Sex				
Male	1 481 353 (39.3)	176 060 (11.9)		1 [Reference]
Female	2 283 007 (60.6)	346 120 (15.2)		1.46 (1.45-1.47)
Race/ethnicity				
Non-Hispanic white	3 053 688 (81.1)	421 615 (13.8)		1 [Reference]
Non-Hispanic black	351 985 (9.3)	61 480 (17.5)		0.83 (0.82-0.84)
Hispanic	198 778 (5.3)	25 877 (13.0)		0.75 (0.74-0.76)
Other	159 910 (4.2)	13 208 (8.3)		0.61 (0.60-0.63)
Dual eligibility				
Yes	1 028 725 (27.3)	231 713 (22.5)		1.38 (1.37-1.39)
No	2 735 635 (72.7)	290 467 (10.6)		1 [Reference]
Comorbidities, No.				
0	1 324 141 (35.2)	98 788 (7.5)		1 [Reference]
1	1 004 673 (26.7)	127 941 (12.7)		1.73 (1.72-1.75)
2	607 431 (16.1)	101 001 (16.6)		2.31 (2.29-2.34)
≥3	828 115 (22.0)	194 450 (23.5)		3.64 (3.61-3.67)
Intraclass correlation coefficient	Null model		Adjusted model	
County level, %	10.90		9.35	

Abbreviations: ESRD, end-stage renal disease; OR, odds ratio.

^a^ Results are from a multilevel analysis, including Medicare enrollees and counties, adjusted for the individual characteristics of the Medicare enrollees listed in the Table, but not including any county characteristics (ie, a null model). Model includes 3118 US counties.

**Figure 1.**
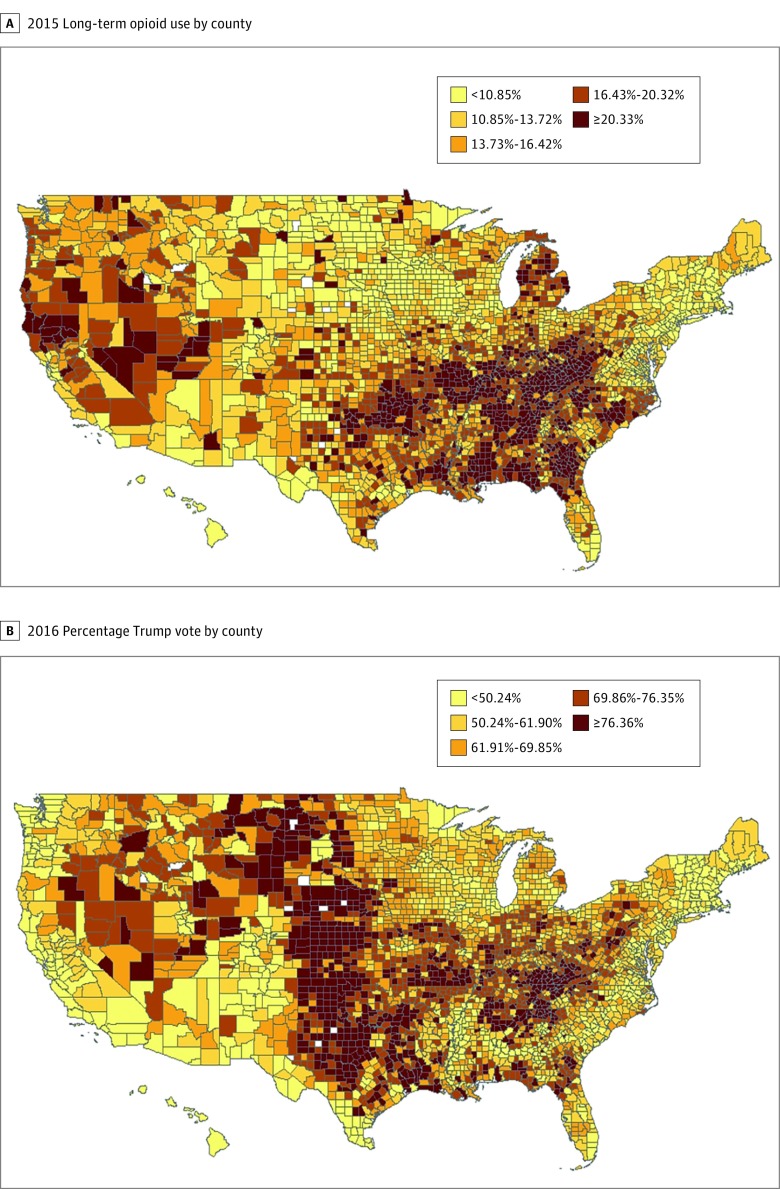
Opioid Use and Voting Patterns by County A, The percentage of Medicare Part D enrollees who received prescriptions for at least a 90-day supply of an opioid in 2015. B, The percentage of the vote for the Republican presidential candidate in 2016. The opioid map includes 3118 of 3142 US counties (99.2%), and the voting map includes 3101 counties (98.7%). In each map, the rates are color coded by quintile of counties. The rates are not adjusted for any individual or county characteristics.

Figure 1 presents 2 maps illustrating opioid use in 3118 of 3142 US counties (99.2%) and 2016 presidential voting patterns in 3101 counties (98.7%). The first map shows the percentage of older Medicare beneficiaries who had an opioid supply covering more than 90 days in 2015, ordered by quintile at the county level. Approximately 1 in 5 counties had long-term opioid prescribing rates greater than 20.10%, while a similar proportion had rates of less than 10.85%. Counties with the highest rates were predominately concentrated in the South and Appalachian areas, as well as Michigan and some western states. The second map shows the percentage of the presidential vote for the Republican candidate for each county, also ordered by quintile. The 2 maps share some similar patterns. The correlation coefficient between the 2 rates at the county level was 0.32 (*P* < .001). Counties in the Great Plains states and also in the Deep South were more likely to be discordant in the 2 measures. The correlation of county-level presidential voting with the percentage of Medicare enrollees who were prescribed insulin was also measured to assess the specificity of the correlation with opioid prescriptions. The correlation was 0.02 (*P* = .17).

Next we examined county-level characteristics associated with chronic opioid use by Medicare enrollees. First, a multilevel analysis was conducted to examine variation in opioid use among counties after controlling for person-level characteristics, including age, sex, race/ethnicity, Medicaid eligibility, number of comorbidities, and whether the Medicare enrollees initially became eligible for Medicare because of disability (Table 1). Female sex, non-Hispanic white race/ethnicity, Medicare coverage for disability or end-stage renal disease, Medicaid eligibility, and increasing number of diagnoses were all associated with increased odds of chronic opioid prescriptions.

**Figure 2.**
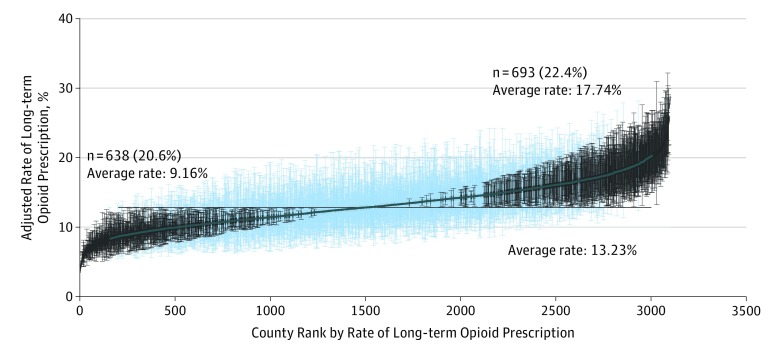
Variation Among US Counties in Adjusted Rates of Chronic Opioid Prescription in 2015 Counties were ranked based on rates from a multilevel model adjusted for patient characteristics included in Table 1. The black horizontal line represents the overall average adjusted rate. Counties with 95% confidence intervals for rates entirely above or below the average adjusted rate are indicated in black. Results are presented for 3100 counties and 3 759 186 enrollees, a 20% national sample of Medicare Part D files.

Figure 2 illustrates the county-level variation in adjusted rates of chronic opioid use. After controlling for individual characteristics, there was still considerable variation in county rates, with 693 of 3100 counties (22.4%) with adjusted rates significantly greater than the mean rate and 638 of 3100 counties (20.6%) with significantly lower rates. The intraclass correlation coefficient for the adjusted model was 0.092, implying that county of residence explained 9.2% of the variation in whether a Medicare recipient received prescriptions for chronic opioid use, independent of the characteristics of the individuals. The correlation between the adjusted county rates of opioid use and the presidential vote was 0.42 (*P* < .001).

Table 2 presents the characteristics of the counties with significantly higher or lower adjusted Republican presidential votes than the average. Most of the socioeconomic variables differed significantly between the 2 sets of counties. The presidential vote was one of the largest differences between counties with high and low rates of opioid use, with the former voting for the Republican candidate at a mean (SE) rate of 59.96% (1.73%) and the latter voting for the Republican candidate at a mean (SE) rate of 38.67% (1.15%). There were also substantial differences in household income, education, racial composition, and the percentage of Medicare enrollees who originally became eligible because of disability. This last difference was unexpected, because Medicare eligibility for disability had been controlled for at the individual level in estimating the adjusted county rates.

**Table 2.** Characteristics Associated With Chronic Opioid Prescriptions for Counties With Significantly Higher Rates Than Average vs Counties With Significantly Lower Rates Than Average

County Characteristic^a^	Mean (SE)			*P* Value
	Total	Lower Opioid Use	Higher Opioid Use	
Counties, No. (%)	3100	638 (20.58)	693 (22.35)	
Rurality, mean (SEM)^b^	2.10 (0.06)	1.54 (0.05)	2.93 (0.15)	<.001
Median household income, $	55 964 (692)	60 577 (1,069)	45 269 (604)	<.001
Adults with high school diploma, %	86.64 (0.38)	87.26 (0.64)	84.32 (0.37)	<.001
Unemployment, %	8.38 (0.12)	8.13 (0.17)	9.48(0.27)	<.001
Republican presidential vote, %	45.92 (0.98)	38.67 (1.15)	59.96 (1.73)	<.001
Married, %	48.18 (0.34)	47.05 (0.48)	49.15 (0.54)	.004
Non-Hispanic white, %	82.17 (0.72)	79.02 (1.13)	85.22 (1.01)	<.001
Male, %	43.70 (0.07)	43.09 (0.09)	44.52 (0.13)	<.001
Original Medicare entitlement for disability, %	23.40 (0.25)	20.76 (0.31)	29.80 (0.38)	<.001
Medicaid eligible, %	18.16 (0.37)	17.86 (0.64)	20.29 (0.38)	.001
Member of HMO, %	28.53 (0.62)	30.54 (1.00)	25.08 (0.84)	<.001
Religious attendance per 1000 population	488.1 (5.3)	490.9 (7.6)	496.3 (11.9)	.70
Single household, %	17.78 (0.24)	17.94 (0.36)	18.43 (0.34)	.32
Part D coverage, %	71.83 (0.25)	72.21 (0.41)	71.77 (0.42)	.46
≥3 Comorbidities, %	23.01 (0.19)	23.60 (0.30)	23.15 (0.30)	.29

Abbreviation: HMO, health maintenance organization.

^a^ Data on counties are from the US Census American Community Survey (ACS) 5-year estimates (2011-2015) and the 20% national sample of Medicare Part D files. The unemployment rates are from the ACS and are higher than estimates produced by the Bureau of Labor Statistics. Individual characteristics of the Medicare enrollees were controlled for when producing the adjusted county rates of long-term opioid prescriptions.

^b^ Rurality is measured by the US Department of Agriculture Rural-Urban Continuum Codes^21^: 1 indicates counties in metropolitan areas of 1 million population or more; 2, counties in metropolitan areas of 250 000 to 1 million population; 3, counties in metropolitan areas of fewer than 250 000 population; 4, urban population of 20 000 or more, adjacent to a metropolitan area; 5, urban population of 20 000 or more, not adjacent to a metropolitan area; 6, urban population of 2500 to 19 999, adjacent to a metropolitan area; 7, urban population of 2500 to 19 999, not adjacent to a metropolitan area; 8, completely rural or less than 2500 urban population, adjacent to a metropolitan area; 9, completely rural or less than 2500 urban population, not adjacent to a metropolitan area.

Next we examined whether the presidential vote was associated with opioid use independent of the demographic and socioeconomic measures. Table 3 presents an analysis showing the percentage of the variation in adjusted county opioid use rates explained by the Republican presidential vote, before and after the addition of other county characteristics. The county opioid use rates were adjusted for differences in individual characteristics among the Medicare recipients. Model 1 includes only the percentage vote for the Republican candidate, which explains 18% of the county-level variation in opioid use. In model 2, all the variables included in Table 2 were added. After controlling for those county characteristics, the presidential vote explained 7% of the variation in opioid use. Model 3 adds variables describing the degree of state regulations on opioid prescribing^22,23,24^; in this model the presidential vote still explains 6% of the county-level variation. The model *R*^2^ was 0.44 for model 2 and 0.46 for model 3, indicating that the variables included explained 44% and 46% of the variance in county opioid rates, respectively. In both models 2 and 3, the presidential vote has the largest explanatory power among county characteristics. Thus, adjusting for county-level socioeconomic measures explained approximately two-thirds of the association between opioid rates and presidential voting rates.

**Table 3.** Socioeconomic and Regulatory Factors Contributing to the Association of the Vote for the Republican Presidential Candidate in 2016 With Rates of Chronic Opioid Prescriptions

Socioeconomic or Regulatory Factor^a^	*R*^2^		Parameter Estimate^b^	Standard Error	*P* Value
	Model	Partial			
Model 1	0.18				
Intercept			7.50	0.23	<.001
% Republican presidential vote		0.18	0.09	0.004	<.001
Model 2	0.44				
Intercept			13.65	1.98	<.001
% Republican presidential vote (each 1% increase)		0.07	0.08	0.005	<.001
% Original entitlement as disabled (1% increase)		0.04	0.12	0.01	<.001
Median household income, per $1000 (1% increase)		0.02	−0.05	0.007	<.001
% Part D coverage (1% increase)		0.02	−0.07	0.009	<.001
Unemployment (1% increase)		0.01	0.13	0.02	<.001
% With ≥3 comorbidities (1% increase)		0.007	−0.05	0.01	<.001
% Adult high school graduate (1% increase)		0.007	−0.05	0.01	<.001
% Married (1% increase)		0.003	0.04	0.01	.001
Rurality^c^ (1 unit increase)		0.003	−0.07	0.02	.004
% Single household (1% increase)		0.002	0.04	0.02	.02
% HMO (1% increase)		0.002	−0.01	0.004	.03
% Non-Hispanic white (1% increase)		0.001	0.009	0.006	.13
% Medicaid eligible (1% increase)		<0.001	−0.01	0.01	.35
% Male (1% increase)		<0.001	0.01	0.02	.46
Religious attendance per 1000 population (1% increase)		<0.001	<.001	<.001	.88
Model 3	0.46				
Intercept			12.05	1.98	<.001
% Republican presidential vote		0.06	0.08	0.005	<.001
% Original entitlement as disabled		0.05	0.12	0.01	<.001
Median household income, per $1000		0.02	−0.05	0.007	<.001
% Part D coverage		0.02	−0.07	0.009	<.001
Unemployment		0.008	0.10	0.02	<.001
% With ≥3 comorbidities		0.009	−0.06	0.01	<.001
% Adult high school graduate		0.004	−0.04	0.01	<.001
% Married		0.003	0.04	0.01	.001
Rurality^c^		<0.001	−0.02	0.03	.32
% Single household		0.003	0.06	0.02	.002
% HMO		<0.001	−0.007	0.004	.13
% White		0.001	0.01	0.006	.04
% Medicaid eligible		<0.001	−0.01	0.01	.26
% Male		<0.001	0.001	0.02	.97
Religious attendance per 1000 population		<0.001	<.001	<.001	.12
Opioid law category, yes vs no^d^					
1		0.007	0.94	0.20	<.001
2		0.003	0.37	0.12	.002
3		0.002	−0.36	0.13	.006
4		0.002	0.25	0.01	.01
5		<0.001	−0.16	0.14	.24
6		<0.001	−0.26	0.32	.42
7		<0.001	0.40	0.34	.25

Abbreviation: HMO, health maintenance organization.

^a^ The county opioid rates are adjusted for the individual characteristics of Medicare enrollees (shown in Table 1). Model 1 includes only the county percentage vote for the Republican presidential candidate. Model 2 adds county-level socioeconomic measures. Model 3 adds whether the state had specific regulations on opioid prescribing.

^b^ Parameter estimate is the change in response (rate of opioid prescriptions) for each 1-unit change in the predictor.

^c^ Rurality is measured by the US Department of Agriculture Rural-Urban Continuum Codes^21^: 1 indicates counties in metropolitan areas of 1 million population or more; 2, counties in metropolitan areas of 250 000 to 1 million population; 3, counties in metropolitan areas of fewer than 250 000 population; 4, urban population of 20 000 or more, adjacent to a metropolitan area; 5, urban population of 20 000 or more, not adjacent to a metropolitan area; 6, urban population of 2500 to 19 999, adjacent to a metropolitan area; 7, urban population of 2500 to 19 999, not adjacent to a metropolitan area; 8, completely rural or less than 2500 urban population, adjacent to a metropolitan area; 9, completely rural or less than 2500 urban population, not adjacent to a metropolitan area.

^d^ The state laws are classified into 7 categories by the Centers for Disease Control and Prevention.^22^ See Methods for description.

The analyses were repeated, using the percentage of county vote for Republican congressional candidates instead of the presidential vote. The correlation with the unadjusted opioid use rates was 0.27 (*P* < .001), and with the adjusted rates it was 0.36 (*P* < .001). The correlation of the county Republican congressional vote with the county presidential vote was high (*r* = 0.82; *P* < .001). The analyses were repeated in Table 3, substituting county Republican congressional vote for the Republican presidential vote. The partial *R*^2^ values for the congressional vote in models 1, 2, and 3 were 0.13, 0.05, and 0.04, respectively, somewhat lower than the values for the presidential Republican vote in Table 3.

## Discussion

In this retrospective study using a national sample of Medicare claims data, chronic use of prescription opioid drugs was correlated with support for the Republican candidate in the 2016 presidential election. Republican support explained 18% of the variance in county rates of opioid use in 3100 counties in the United States, with counties whose opioid prescription rates were above average having a higher mean (SE) Republican vote (59.96% [1.73%]) than counties with prescription rates below average (38.67% [1.15%]). This association is related to underlying county socioeconomic characteristics that are common to both chronic opioid use and voting patterns, particularly characteristics pertaining to income, disability, insurance coverage, and unemployment.

The findings of this study add to the emerging literature on the relationship between health status and support of Donald Trump in the 2016 election. Wasfy and colleagues^25^ examined the difference in support for Trump in 2016 and for Mitt Romney, the Republican candidate in 2012, among 3009 counties. Eighty-eight percent of counties had a net voting shift toward the Republican candidate in 2016. They then examined the relationship between voting shift and a 7-item measure of unhealthfulness. The unhealthfulness score accounted for 68% of the variance in the magnitude of the voting shift. Similarly, Bor^26^ reported that a 2016 Republican presidential vote at the county level was strongly and negatively correlated with change in life expectancy between 2008 and 2016, and Monnat^13^ reported that counties with drug, alcohol, and suicide mortality rates above the median showed heavier support for Trump in 2016 than for Romney in 2012. In many areas with high rates of drug overdose, voter turnout in 2016 exceeded that in 2012, with Donald Trump overwhelmingly favored.^14^

The current study and the other studies discussed were ecological, measuring associations at a county level between the presidential vote and health indicators. There is some evidence that the association is indeed contextual. An analysis of interviews with supporters of President Trump conducted by Gallup concluded that they came from areas where residents have high rates of poor health and lack of upward mobility, even if the health and economic status of the individual respondents were good.^27^ The community context seemed at least as strong an influence as individual economic factors.^27,28^ An analogous finding of contextual effects on opioid prescriptions was shown in the current analysis. In both Table 2 and Table 3, the county rate of Medicare recipients originally enrolled for disability was associated with adjusted county opioid use rate, even though the county opioid use rates were adjusted for disability at the individual level.

### Limitations

There are several limitations to our analysis. The county presidential vote is from 2016 and includes all voters, while the information on prolonged opioid prescriptions was from 2015 and was generated only from Medicare Part D enrollees, approximately 72% of the entire Medicare population.^20^ In addition, prescription opioids are only part of the opioid epidemic, accounting for approximately half of opioid-related deaths.^2^ The characteristics of the prescribers of the opioids were not examined, although prescriber behavior clearly plays an important role. As noted previously, the analyses are ecological, linking opioid use and voting at the county, and not the individual, level. Approximately two-thirds of the association between opioid rates and presidential voting was explained by socioeconomic variables. The socioeconomic variables were limited to the available data. Our assumption is that all of the association between opioid use and voting patterns is explainable by socioeconomic, legal, environmental, and cultural factors, but that assumption cannot be tested with the current data.

## Conclusions

Experts have struggled to explain both the root causes of the opioid epidemic and the results of the 2016 election. As noted by Mayhew, “in…periods of populist anger the causes of that anger are hard to explain using standard measures of economic well-being.”^28^

Many studies have shown that the relationship between health and social variables (such as employment status, income, and neighborhood) is at least as strong as the relationship between health and biological variables.^29,30^ Public health policy directed at stemming the opioid epidemic must go beyond the medical model and incorporate socioenvironmental disadvantage factors and health behaviors into policy planning and implementation.^30,31^
